# Evaluation of at-home physiotherapy: machine-learning prediction with smart watch inertial sensors

**DOI:** 10.1302/2046-3758.123.BJR-2022-0126.R1

**Published:** 2023-03-01

**Authors:** Philip Boyer, David Burns, Cari Whyne

**Affiliations:** 1 Institute of Biomedical Engineering, University of Toronto, Toronto, Canada; 2 Sunnybrook Research Institute, Toronto, Canada; 3 Harborview Medical Center, Seattle, Washington, USA; 4 University of Washington, Seattle, Washington, USA; 5 Division of Orthopaedic Surgery, Department of Surgery, University of Toronto, Toronto, Canada

**Keywords:** Physiotherapy, Physical therapy, Rehabilitation, Inertial measurement units, Machine learning, physiotherapy, shoulder, rotator cuff injuries, physiotherapists, rotator cuff, accelerometer, Full-thickness rotator cuff tears, variances, standard deviation, flexion

## Abstract

**Aims:**

An objective technological solution for tracking adherence to at-home shoulder physiotherapy is important for improving patient engagement and rehabilitation outcomes, but remains a significant challenge. The aim of this research was to evaluate performance of machine-learning (ML) methodologies for detecting and classifying inertial data collected during in-clinic and at-home shoulder physiotherapy exercise.

**Methods:**

A smartwatch was used to collect inertial data from 42 patients performing shoulder physiotherapy exercises for rotator cuff injuries in both in-clinic and at-home settings. A two-stage ML approach was used to detect out-of-distribution (OOD) data (to remove non-exercise data) and subsequently for classification of exercises. We evaluated the performance impact of grouping exercises by motion type, inclusion of non-exercise data for algorithm training, and a patient-specific approach to exercise classification. Algorithm performance was evaluated using both in-clinic and at-home data.

**Results:**

The patient-specific approach with engineered features achieved the highest in-clinic performance for differentiating physiotherapy exercise from non-exercise activity (area under the receiver operating characteristic (AUROC) = 0.924). Including non-exercise data in algorithm training further improved classifier performance (random forest, AUROC = 0.985). The highest accuracy achieved for classifying individual in-clinic exercises was 0.903, using a patient-specific method with deep neural network model extracted features. Grouping exercises by motion type improved exercise classification. For at-home data, OOD detection yielded similar performance with the non-exercise data in the algorithm training (fully convolutional network AUROC = 0.919).

**Conclusion:**

Including non-exercise data in algorithm training improves detection of exercises. A patient-specific approach leveraging data from earlier patient-supervised sessions should be considered but is highly dependent on per-patient data quality.

Cite this article: *Bone Joint Res* 2023;12(3):165–177.

## Article focus

- This research evaluates the performance of machine-learning (ML) algorithms in predicting and classifying shoulder physiotherapy exercises from inertial data captured with commercial smartwatches worn by patients as they performed exercises in the supervised clinical and unsupervised at-home settings.

## Key messages

- Detection of non-exercise data in the home setting can be improved by including a proxy activities of daily living dataset in algorithm training, by grouping exercises by similar motion for classification, and by leveraging patient-specific exercise data from in-clinic visits.
- This research highlights the importance of verifying ML algorithm performance, not only in the lab or clinical setting but also with patients in the at-home environment.

## Strengths and limitations

- Algorithm performance in classifying physiotherapy exercise performance was evaluated using real-world clinical data collected via a commercial smartwatch from patients being treated for rotator cuff pathology both in the clinic and the at-home setting.
- Ground truth labels for the independent at-home exercises (in-distribution vs out-of-distribution) were assigned manually after data collection, leading to potential uncertainty in the assigned labels.

## Introduction

Shoulder pain affects approximately 16% to 26% of the adult population,^1-3^ and can significantly impact quality of life.^4-6^ Rotator cuff pathology is the most common condition affecting between 60% and 85% of patients with shoulder disorders.^2,3^ Physiotherapy is a first-line treatment for rotator cuff pathology which can improve patient outcomes (pain and function).^7,8^ A large proportion of prescribed physiotherapy is expected to be performed independently in the at-home setting, and adherence to assigned home exercise is considered important to programme effectiveness.^9-11^ Accurate and objective measurement of adherence is critical to identifying and addressing barriers to exercise. Low-tech solutions (i.e. patient-reported diaries) have low completion rates and are subject to various biases.^12,13^ Wearable devices containing inertial measurement units (IMUs) have gained wide acceptance in human activity tracking, including physiotherapy participation.

Machine-learning (ML) algorithms have been developed for time series data enabling the analysis of IMU data generated from wearables.^14,15^ In using ML to evaluate physiotherapy participation from IMU data, it is important to accurately classify exercises (in-distribution data) and identify periods when participants may be performing unrelated tasks (out-of-distribution data (OOD).^16,17^ OOD data may negatively impact ML classifier accuracy, and thus should be removed in pre-processing (before performing exercise classification) or accounted for through use of an open set classifier.

Systems proposed for exercise classification in the home setting using IMUs or image-based motion tracking, with statistical or ML-based classification algorithms,^18-21^ are often solely tested in laboratory or in-clinic environments. Performance of exercises and OOD activities may be much more variable in the at-home environment. Recent work measuring at-home exercise participation with IMUs and ML classification in stroke patients has noted limitations with regard to the lack of validation on at-home data.^21^ Lack of direct analysis of at-home exercise and OOD data may limit the ML-derived accuracy of physiotherapy adherence.^22^

We have previously reported on using smartwatch inertial data for classifying shoulder physiotherapy exercise in healthy volunteers.^23^ Our ML physiotherapy prediction system was further evaluated with a clinical dataset collected from 42 patients undergoing shoulder physiotherapy for rotator cuff pathology.^24^ With our system, a record of inertial data is generated whenever a patient wears their watch (in-clinic or at-home). In-clinic data are labelled in real time by physiotherapists via a companion app (tablet interface). Data collected at home are analyzed with a ML pipeline, where non-exercise data are removed by a trained OOD detector and a separate classifier labels exercises in the remaining data.

This study aims to assess the performance of our system in detecting and classifying physiotherapy exercises in the unsupervised at-home setting, considering: exercise groupings, using a proxy OOD dataset in training, and leveraging historic patient-specific in-clinic data.

## Methods

### Datasets

Inertial data (accelerometer, gyroscope) were collected from 42 patients with rotator cuff pathology wearing Huawei Watch 2 smartwatches (Huawei Technologies, China) while they performed prescribed physiotherapy exercises in clinic under physiotherapist supervision (1,951 records, 23.8 hours, labelled by exercise class (n = 18, Table I)), and independently at home (3,659 records, 1,288 hours, unlabelled) (REB #353-2018).^24^ An additional OOD class was drawn from the publicly available dataset ProxyA,^25^ consisting of inertial data collected from 20 healthy adults performing activities of daily living (ADLs).

**Table I.** Physiotherapy exercises included in the dataset and motion groupings.

Exercise	Count	Motion	Simple motion	Position
Active shoulder flexion	100	Flexion	Elevation	Upright
Active shoulder abduction	35	Abduction	Elevation	Upright
Assisted shoulder flexion	119	Flexion	Elevation	Upright
Assisted shoulder flexion	87	Flexion	Elevation	Lying
Shoulder girdle stabilization with elevation	130	Flexion	Elevation	Upright
Assisted shoulder external rotation	89	External rotation	Rotation	Upright
Assisted shoulder internal rotation	96	Internal rotation	Rotation	Upright
Assisted shoulder internal rotation	61	Internal rotation	Rotation	Side-lying
Resisted shoulder internal rotation	131	Internal rotation	Rotation	Upright, adducted
Resisted shoulder external rotation	163	External rotation	Rotation	Upright, adducted
Resisted shoulder external rotation	20	External rotation	Rotation	Upright, abducted
Resisted row	206	Row	Row	Bent over
Resisted triceps pull down	67	Elbow-extension	Elbow-flexion	Upright
Resisted lat pull down	194	Pull down	Pull down	Upright
Resisted lat pull down (external rotation)	1	Pull down	Pull down	Upright
Press up against wall	133	Press up	Press up	Upright
Resisted seratus anterior	2	Press up	Press up	Upright
Push up	3	Press up	Press up	Prone

‘Motion position’ and ‘simple motion position’ groupings are the same categories as listed for motion and simple motion, but further grouped by position information, e.g. motion position grouping of active shoulder flexion is flexion-upright, whereas simple motion position is elevation-upright.

Patients attended supervised clinical physiotherapy sessions at one-week intervals to a maximum of 12 weeks. Mean age of patients was 45 years (standard deviation (SD) 13), with 15 males and 27 females. Full-thickness rotator cuff tears were present in 13 patients, 12 patients had partial-thickness tears, and 17 patients had no tear. All patients were treated non-surgically. At baseline, patients had mean Disability of the Arm, Shoulder and Hand questionnaire (DASH) scores of 44 (SD 21) and mean numerical pain rating scale (NPRS) scores of 5.2 (SD 1.9).

### Data processing

The Android Wear OS samples the inertial sensors asynchronously and irregularly (~50 Hz). Data were interpolated to yield consistent 50 Hz time steps. A sliding window segmentation with ten-second window length and step size of 50 was used to tensorize and augment the dataset (Seglearn).^14^

### Feature extraction

Engineered statistical features were calculated for each sensor channel on each window segment including: median, absolute energy (root mean squared), SD, variance, minimum, maximum, skewness, kurtosis, mean spectral energy, and mean crossings. Deep feature extraction was performed using the output of the penultimate layer of a fully convolutional network (FCN) (i.e. pre-Softmax).

### Machine-learning pipeline

**Fig. 1:**
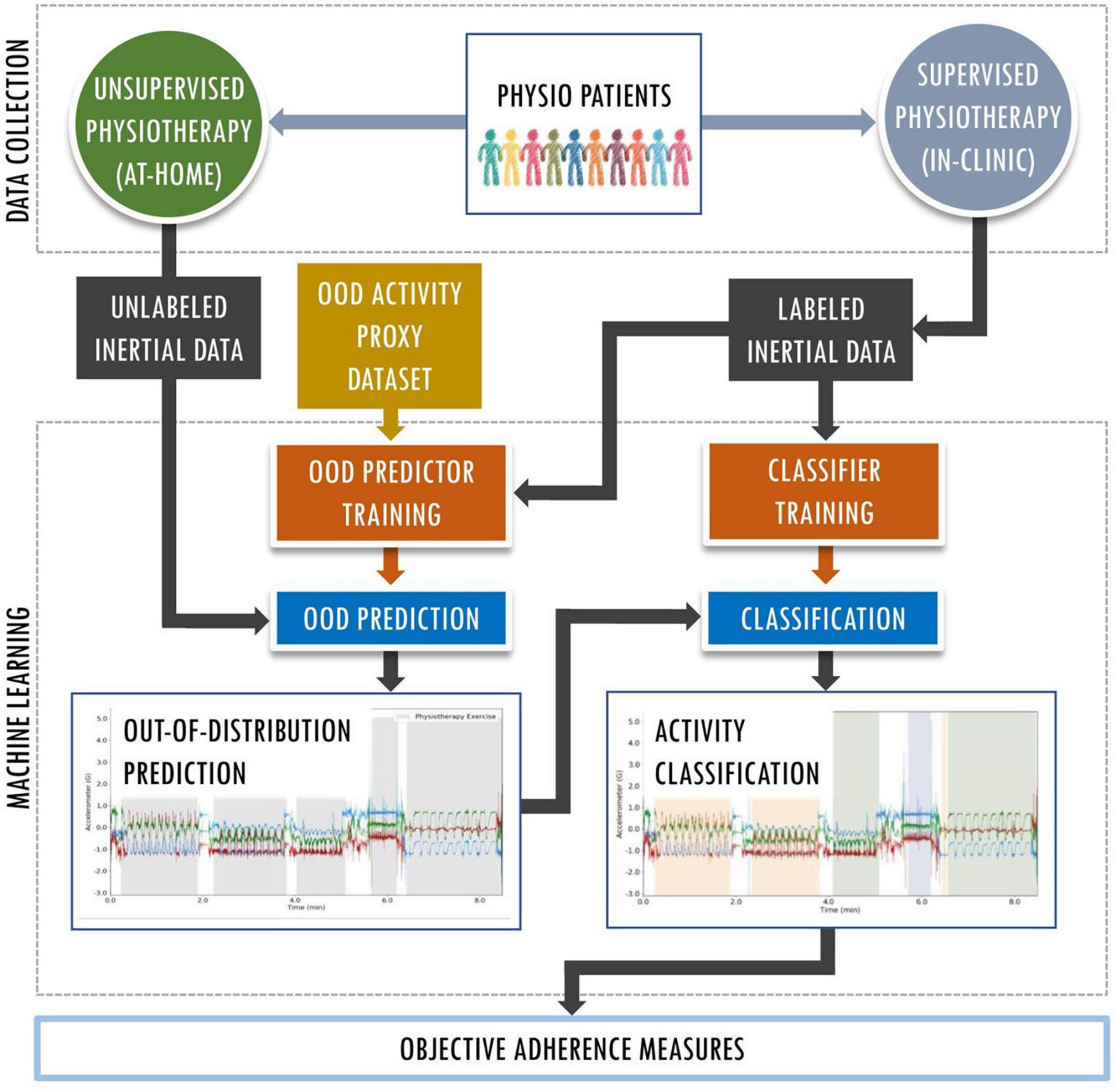
(System) data collection and machine-learning pipeline for identification and classification of at-home shoulder physiotherapy exercises. OOD, out-of-distribution.

The ML pipeline operates in a two-stage process: labelled inertial data were first used to train an OOD detector for removal of non-exercise data, and then to train a model for classification of exercises (Figure 1).

### Algorithms

Algorithms were selected for evaluation based on performance (> 90% classification accuracy, > 0.90 area under the receiver operating characteristic (AUROC)) on shoulder physiotherapy IMU data collected from healthy subjects and on ADL datasets.^23,26,27^ These algorithms are commonly employed where inertial data are used to predict physiotherapy and rehabilitation exercise with good performance.^21,28^

### Patient-specific data

**Fig. 2:**
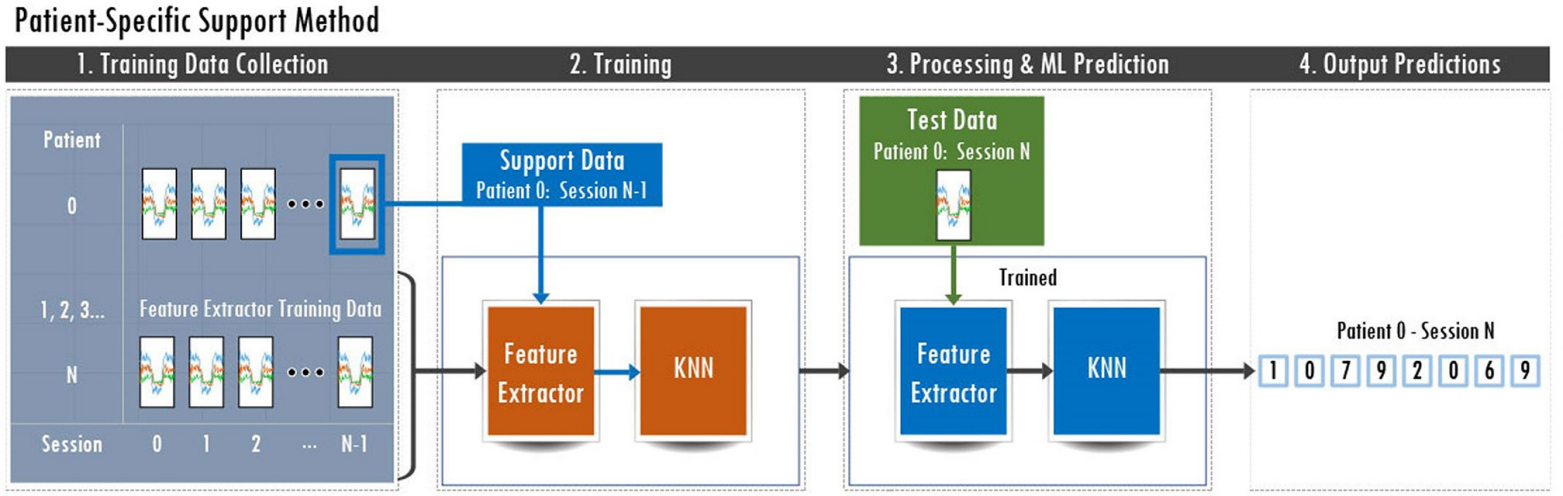
Patient-specific support method. In stage 1, Patient 0 is in the test split of the cross-validation fold. In stage 2, a feature extractor (e.g. fully convolutional network) is trained on the training split, while the last test fold session is used as ‘support’ data to train a K-Nearest Neighbour (KNN) algorithm on this more limited, but patient-specific, distribution. In stage 3, the data of the patient’s test session are transformed by the trained feature extractor and passed as input to the trained KNN algorithm to output the predictions of stage 4.

The patient-specific method (Figure 2) uses a K-Nearest Neighbour (KNN) algorithm trained on the last supervised session of each exercise on a per-patient basis. Input to the KNN was either engineered statistical features or FCN embeddings, with either feature extractor fit on the training dataset. The Scikit-learn implementation of KNN was used with three nearest neighbours and a Euclidean distance metric.^29^ Otherwise, default hyperparameters were used.

### Out-of-distribution detection algorithms

OOD detection algorithms were as follows: 1) KMeans (Engineered Features/FCN Embeddings) - Unsupervised algorithm with iterative updating assigning data to clusters. OOD data detected by comparing distance to cluster centre versus threshold value. Scikit-Learn implementation with default hyperparameters;^29^ 2) FCN Softmax Threshold - Softmax layer output in a deep learning (DL) model is often interpreted as a confidence metric;^30^ samples below threshold may be classified as OOD; and 3) patient-specific method (Engineered Features/FCN Embeddings) - OOD prediction by KNN is based on exceeding a threshold mean distance from nearest n-neighbors (n = 3) in the support set (most recent in-clinic session). OOD thresholds were determined based on a minimum desired sensitivity of 0.90 to reduce likelihood of exercise non-detection (Type II errors).

### Out-of-distribution detection algorithms with proxy dataset

Including proxy OOD data (SPARS9x-OOD, unlabelled ADL data matched to size of in-distribution dataset) as an additional class in training converts the OOD problem to supervised classification.^26^

### In-distribution classification algorithms

In-distribution classification algorithms were as follows: 1) FCN supervised - a DL model used for time series classification (batch size = 256, learning rate = 0.0001, epochs = 150);^31^ Keras implementation available online;^32^ 2) random forest (Engineered Features/FCN Embeddings) - ensemble of decision trees, predicts based on majority vote; Scikit-Learn implementation with default hyperparameters;^29^ and 3) patient-specific method (Engineered Features/FCN Embeddings) - for in-distribution classification, KNN predicts class directly based on support.

### Validation

For labelled in-clinic data, algorithm performance was evaluated using five-fold cross-validation, splitting the dataset by study subject. All classes (exercises, Table I) were represented in each cross-validated run of the model. The test sets were composed of the most recent session of labelled in-clinic data in the test split of each fold. The second-to-last session was used for patient-specific methods. Those records that did not have supporting data of the same class from previous in-clinic sessions were excluded to ensure consistency in test sets throughout the analysis. As data were only labelled while patients performed physiotherapy exercises in-clinic, a size-matched proxy non-exercise activity class was used (ProxyA-OOD). The output was a binary prediction (exercise/non-exercise). A range of ‘exercise groupings’ were evaluated (Table I).

Validation of OOD detection techniques was also performed on the unlabelled at-home dataset (five-fold cross-validation, splitting the dataset by study subject). Each OOD method was evaluated on a total of 25 randomly selected records from five patients. Manual segmentation was performed to identify ground truth exercise periods. Four metrics were used to analyze results: AUROC, F1 score, sensitivity, and specificity. The F1 score is the harmonic mean of precision and recall, and thus is a more suitable metric for evaluation than accuracy in cases where there is an unbalanced dataset in terms of class representation. Records found to not include any exercises during the manual segmentation process were excluded from F1 score and sensitivity calculations. The effect of exercise groupings was also evaluated, but in-distribution classification accuracy was not assessed for the unlabelled at-home dataset as specific exercise class cannot be determined visually.

## Results

### Labelled in-clinic data

**Fig. 3:**
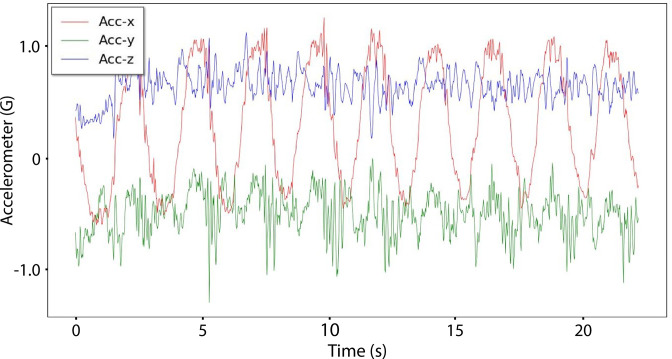
Sample accelerometer inertial data of resisted triceps pull down (standing) in the labelled test set collected in clinic by Patient P3. Acc-x, Acc-y, and Acc-z indicate acceleration in x, y, and z axes of the accelerometer.

Among non-proxy methods for OOD detection (Table II), patient-specific with engineered features performed best (AUROC = 0.924). Patient P3 had a more limited AUROC (0.830) with this method; seven of nine test records from P3 exhibited repetitive motion (e.g. Figure 3), but two did not (e.g. Supplementary Figure a), possibly indicating mistakenly collected or mislabelled recordings. Removing these two records from the test set raised the AUROC for this patient (0.938). Among proxy methods (Table III), random forest with engineered features performed best overall (AUROC = 0.985). Motion type groupings did not consistently improve OOD prediction accuracy across algorithms.

**Table II.** Out-of-distribution detection area under the receiver operating characteristic – no proxy in training.

Grouping	KMeans - engineered	KMeans - embedding	FCN Softmax threshold	Patient-specific - engineered	Patient-specific - embedding
Exercise	0.849 (0.017)	0.820 (0.019)	0.605 (0.033)	0.924 (0.014)*	0.914 (0.017)†
Motion	0.839 (0.020)	0.817 (0.023)	0.634 (0.036)	0.924 (0.014)*	0.905 (0.015)
Motion position	0.850 (0.018)	0.824 (0.019)	0.622 (0.020)	0.924 (0.014)*	0.910 (0.017)†
Simple motion	0.836 (0.022)	0.782 (0.031)	0.709 (0.035)	0.924 (0.014)*	0.884 (0.014)
Simple motion position	0.848 (0.018)	0.816 (0.040)	0.670 (0.028)	0.924 (0.014)*	0.902 (0.018)

Mean cross-validation AUROC with standard error in brackets for labelled in-clinic dataset.

* Highest AUROC obtained.

† Within standard error of highest AUROC result.

AUROC, area under the receiver operating characteristic; FCN, fully convolutional network.

**Table III.** Out-of-distribution detection area under the receiver operating characteristic – supervised classification via proxy out-of-distribution class in training.

Grouping	Random forest - engineered	Random forest - embedding	FCN supervised	Patient-specific - engineered	Patient-specific - embedding
Exercise	0.984 (0.005)*	0.969 (0.009)	0.967 (0.010)	0.936 (0.011)	0.962 (0.013)
Motion	0.985 (0.005)†	0.971 (0.008)	0.972 (0.008)	0.937 (0.011)	0.961 (0.013)
Motion position	0.985 (0.005)†	0.969 (0.007)	0.973 (0.007)	0.936 (0.011)	0.957 (0.014)
Simple motion	0.985 (0.005)†	0.973 (0.006)	0.971 (0.009)	0.937 (0.011)	0.962 (0.012)
Simple motion position	0.985 (0.005)†	0.971 (0.006)	0.965 (0.007)	0.936 (0.011)	0.964 (0.012)

Mean cross-validation AUROC with standard error in brackets for labelled in-clinic dataset.

* Within standard error of highest AUROC result.

† Highest AUROC obtained.

AUROC, area under the receiver operating characteristic; FCN, fully convolutional network.

Simple motion grouping yielded the best in-distribution classification performance (Table IV), with the highest accuracy (0.903) achieved by the patient-specific method with FCN embeddings. Use of FCN embeddings yielded an improvement over engineered features for the patient-specific method.

**Table IV.** In-distribution classification accuracy results.

Grouping	Random forest - engineered	Random forest - embedding	FCN supervised	Patient-specific - engineered	Patient-specific - embedding
Exercise	0.648 (0.030)	0.643 (0.043)	0.650 (0.035)	0.762 (0.040)	0.801 (0.028)
Motion	0.767 (0.020)	0.781 (0.028)	0.792 (0.020)	0.804 (0.031)	0.850 (0.018)
Motion position	0.683 (0.032)	0.699 (0.039)	0.703 (0.041)	0.780 (0.034)	0.802 (0.026)
Simple motion	0.865 (0.022)	0.871 (0.023)	0.883 (0.019)	0.849 (0.030)	0.903 (0.017)*
Simple motion position	0.740 (0.031)	0.750 (0.043)	0.769 (0.039)	0.797 (0.036)	0.822 (0.032)

Mean cross-validation accuracy with standard error in brackets for labelled in-clinic dataset.

* Highest accuracy obtained.

FCN, fully convolutional network.

### Unlabelled at-home data

Analysis results for the at-home dataset are shown in Table V (no proxy in training) and Table VI (proxy in training). Training with the proxy dataset provided more robust identification of ‘low-motion’ or rest activities between exercises, with mean specificity increasing from 0.507 (non-proxy methods) to 0.846. Of those proxy methods, FCN-supervised grouped by exercise achieved the highest AUROC (0.919), although random forest results are within the standard error.

**Table V.** Unlabelled at-home dataset out-of-distribution detection results - no proxy in training. Scores shown as mean of cross-validation with standard error in brackets.

Experiment	Classification	AUROC	F1 Score	Sensitivity	Specificity
KMeans – Embedding	Exercise	0.744 (0.049)	0.728 (0.058)	0.882 (0.050)	0.405 (0.071)
	Motion	0.758 (0.045)	0.725 (0.046)	0.838 (0.053)	0.506 (0.072)
	Motion position	0.755 (0.035)	0.711 (0.076)	0.866 (0.054)	0.414 (0.028)
	Simple motion	0.703 (0.009)	0.708 (0.055)	0.838 (0.051)	0.387 (0.062)
	Simple motion position	0.777 (0.041)*	0.734 (0.048)	0.877 (0.036)	0.477 (0.077)
KMeans – Engineered	Exercise	0.682 (0.088)	0.717 (0.072)	0.734 (0.072)	0.519 (0.075)
	Motion	0.680 (0.086)	0.728 (0.070)	0.762 (0.072)	0.488 (0.067)
	Motion position	0.685 (0.086)	0.721 (0.070)	0.740 (0.074)	0.504 (0.068)
	Simple motion	0.680 (0.087)	0.722 (0.075)	0.753 (0.080)	0.488 (0.064)
	Simple motion position	0.688 (0.088)	0.726 (0.075)	0.749 (0.076)	0.517 (0.073)
FCN Softmax threshold	Exercise	0.665 (0.034)	0.724 (0.058)	0.846 (0.046)	0.363 (0.062)
	Motion	0.762 (0.049)	0.739 (0.056)	0.878 (0.039)	0.391 (0.069)
	Motion position	0.721 (0.045)	0.729 (0.063)	0.863 (0.038)	0.372 (0.059)
	Simple motion	0.765 (0.030)	0.742 (0.045)	0.858 (0.023)	0.447 (0.037)
	Simple motion position	0.723 (0.041)	0.739 (0.050)	0.856 (0.035)	0.410 (0.058)
Patient-specific – Engineered	Exercise	0.770 (0.082)*	0.751 (0.064)	0.728 (0.089)	0.702 (0.072)
	Motion	0.770 (0.082)*	0.751 (0.064)	0.728 (0.089)	0.702 (0.072)
	Motion position	0.770 (0.082)*	0.751 (0.064)	0.728 (0.089)	0.702 (0.072)
	Simple motion	0.770 (0.082)*	0.751 (0.064)	0.728 (0.089)	0.702 (0.072)
	Simple motion position	0.770 (0.082)*	0.751 (0.064)	0.728 (0.089)	0.702 (0.072)
Patient-specific – Embedding	Exercise	0.807 (0.056)†	0.682 (0.051)	0.796 (0.088)	0.479 (0.158)
	Motion	0.788 (0.051)*	0.688 (0.050)	0.779 (0.085)	0.547 (0.140)
	Motion position	0.775 (0.056)*	0.668 (0.058)	0.778 (0.093)	0.444 (0.126)
	Simple motion	0.777 (0.048)*	0.673 (0.062)	0.746 (0.109)	0.511 (0.157)
	Simple motion position	0.803 (0.058)*	0.686 (0.053)	0.803 (0.086)	0.495 (0.140)

Performance of algorithm prediction versus manually labelled ground truth for at-home dataset.

* Within standard error of highest value obtained.

† Highest value obtained.

AUROC, area under the receiver operating characteristic; FCN, fully convolutional network.

**Table VI.** Unlabelled at-home dataset out-of-distribution detection results - proxy in training. Scores shown as mean of cross-validation with standard error in brackets.

Experiment	Classifcation	AUROC	F1 Score	Sensitivity	Specificity
Random forest – Embedding	Exercise	0.918 (0.028)*	0.824 (0.061)	0.859 (0.065)	0.811 (0.013)
	Motion	0.918 (0.029)*	0.827 (0.058)	0.858 (0.061)	0.826 (0.016)
	Motion position	0.916 (0.029)*	0.832 (0.060)	0.866 (0.066)	0.826 (0.018)
	Simple motion	0.916 (0.028)*	0.824 (0.058)	0.857 (0.067)	0.805 (0.014)
	Simple motion position	0.913 (0.029)*	0.814 (0.066)	0.841 (0.076)	0.810 (0.012)
Random forest – Engineered	Exercise	0.904 (0.019)*	0.788 (0.050)	0.714 (0.056)	0.903 (0.014)
	Motion	0.904 (0.019)*	0.796 (0.058)	0.736 (0.051)	0.889 (0.019)
	Motion position	0.902 (0.020)*	0.798 (0.045)	0.732 (0.050)	0.900 (0.018)
	Simple motion	0.906 (0.018)*	0.806 (0.043)	0.752 (0.046)	0.879 (0.020)
	Simple motion position	0.904 (0.019)*	0.798 (0.046)	0.737 (0.050)	0.890 (0.019)
FCN supervised	Exercise	0.919 (0.035)†	0.812 (0.066)	0.821 (0.079)	0.853 (0.018)
	Motion	0.917 (0.035)*	0.813 (0.064)	0.854 (0.072)	0.807 (0.027)
	Motion position	0.915 (0.034)*	0.820 (0.060)	0.849 (0.061)	0.823 (0.025)
	Simple motion	0.902 (0.040)*	0.811 (0.067)	0.849 (0.065)	0.779 (0.023)
	Simple motion position	0.915 (0.035)*	0.822 (0.062)	0.854 (0.060)	0.817 (0.023)
Patient-specific – Engineered	Exercise	0.825 (0.044)	0.771 (0.064)	0.750 (0.063)	0.828 (0.050)
	Motion	0.825 (0.044)	0.772 (0.063)	0.750 (0.063)	0.828 (0.050)
	Motion position	0.825 (0.044)	0.772 (0.063)	0.750 (0.063)	0.828 (0.050)
	Simple motion	0.825 (0.044)	0.772 (0.063)	0.750 (0.063)	0.827 (0.050)
	Simple motion position	0.825 (0.044)	0.772 (0.063)	0.750 (0.063)	0.827 (0.050)
Patient-specific – Embedding	Exercise	0.818 (0.046)	0.774 (0.056)	0.722 (0.075)	0.882 (0.048)
	Motion	0.814 (0.047)	0.765 (0.059)	0.713 (0.082)	0.873 (0.046)
	Motion position	0.840 (0.042)	0.801 (0.048)	0.766 (0.063)	0.882 (0.050)
	Simple motion	0.834 (0.040)	0.778 (0.061)	0.737 (0.073)	0.873 (0.034)
	Simple motion position	0.842 (0.054)	0.793 (0.072)	0.752 (0.088)	0.894 (0.035)

Performance of algorithm prediction versus manually labelled ground truth for at-home dataset.

* Standard error of highest value obtained.

† Highest value obtained.

AUROC, area under the receiver operating characteristic; FCN, fully convolutional network.

**Fig. 4:**
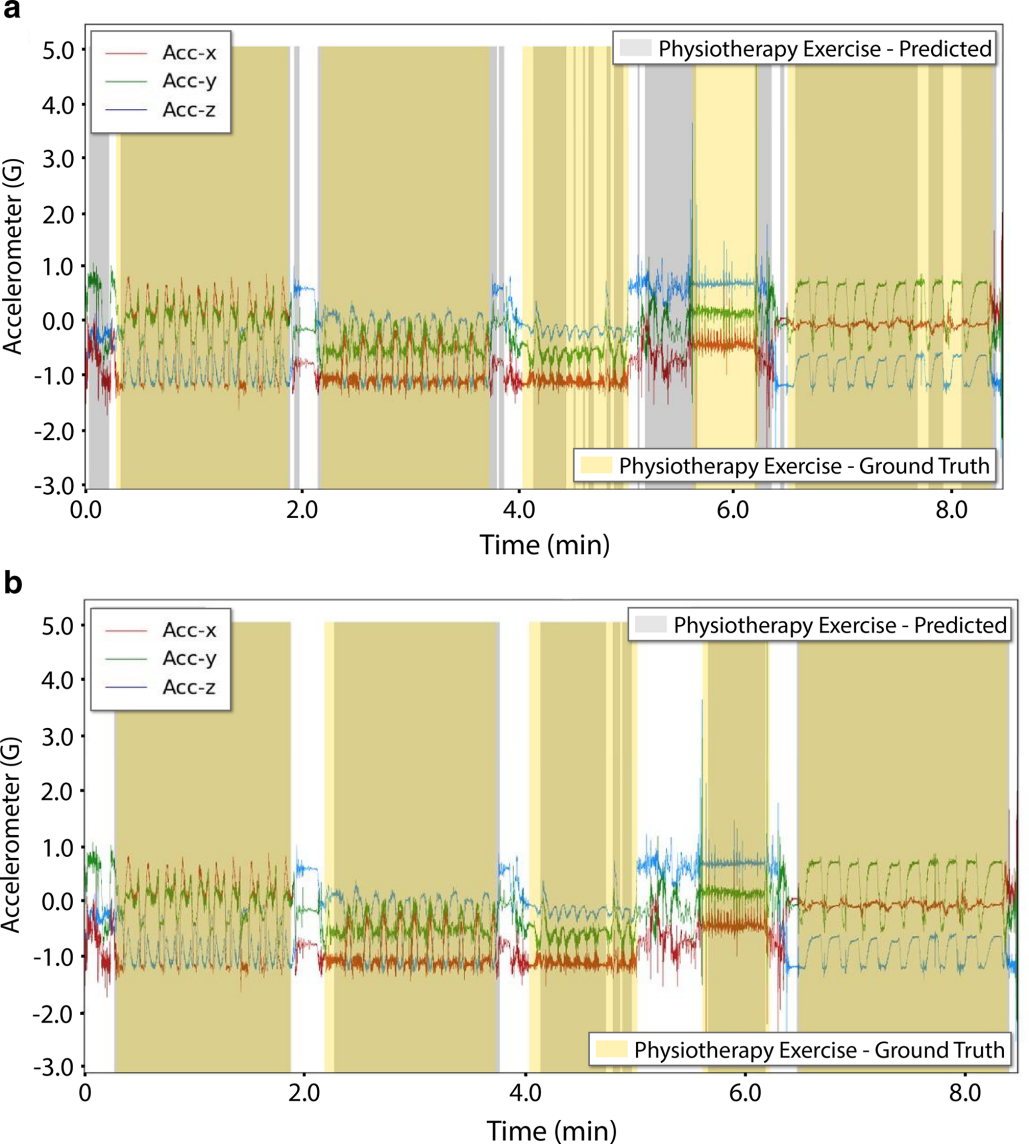
Sample performance of out-of-distribution (OOD) prediction of at-home physiotherapy exercise activity on two records of accelerometer inertial data from patient P0 with a) patient-specific support method with engineered features (without proxy dataset) (area under the receiver operating characteristic (AUROC) 0.907), and b) random forest – fully convolutional network embeddings with OOD proxy dataset in the training set (AUROC 0.968). The misclassified exercise shown in a) exhibits very little motion relative to the other exercises in that record. Improvements from including proxy in training were similar for all algorithms.

In example record Figure 4a, FCN Softmax without proxy commits a Type II error, whereas FCN supervised in Figure 4b correctly predicts the low-motion exercise. These examples are representative of the overall impact of including proxy data as an additional training class.

**Fig. 5:**
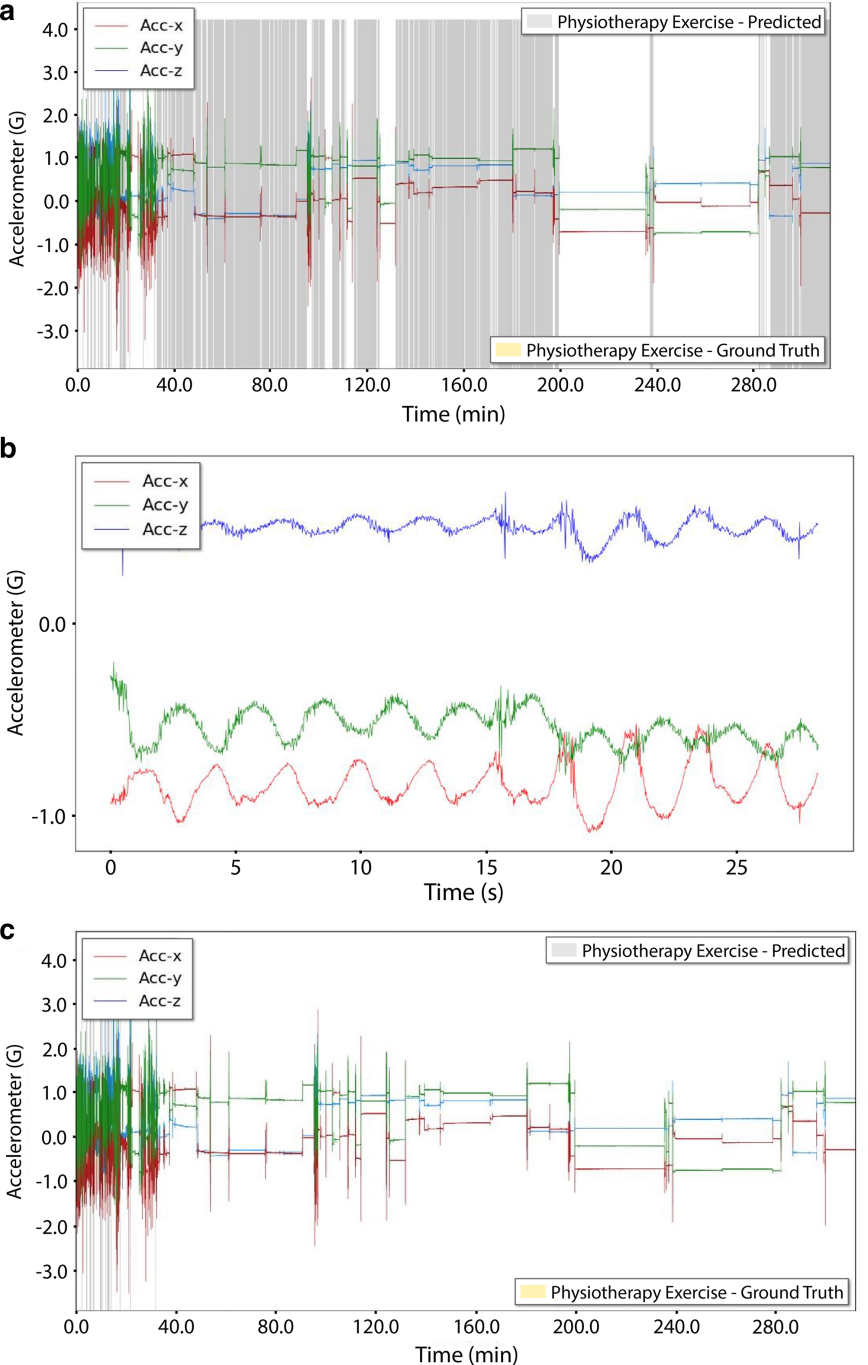
a) This record shows > five hours of data that appear to consist entirely of non-exercise, most of which is incorrectly predicted as exercise by the patient-specific method with engineered features without proxy (specificity 0.445). When the supervised labelled press-up record of b) is removed from the support set for this record, the resulting improvement is shown in c) (specificity 0.985). Note that it was not only this particular press-up record that caused this issue, but also any previous press-up exercises that the patient performed in clinic at earlier dates that were substituted in support.

Table VII summarizes results of two algorithms by patient. Patients P0 and P4 in particular had minimal periods of non-exercise and performed at-home exercises similarly to in-clinic (Figure 4). In contrast, patient P3 collected lengthy periods of non-exercise (Figure 5a) which was predicted as press-up (specificity = 0.445). Removing the P3 press-up record from the support set (Figure 5b) resulted in improved specificity (0.985, Figure 5c). However, this reduced exercise detection sensitivity for this patient from 0.404 to 0.328. The FCN-supervised with proxy method performed similarly to the labelled in-clinic analysis in 3/5 patients.

**Table VII.** Unlabelled at-home dataset – by patient (area under the receiver operating characteristic).

**Patient**	**Patient-specific engineered features – no proxy in training**	**FCN supervised – proxy in training**
P0	0.913	0.979
P1	0.838	0.947
P2	0.761	0.794
P3	0.458	0.896
P4	0.878	0.979

FCN, fully convolutional network.

**Fig. 6:**
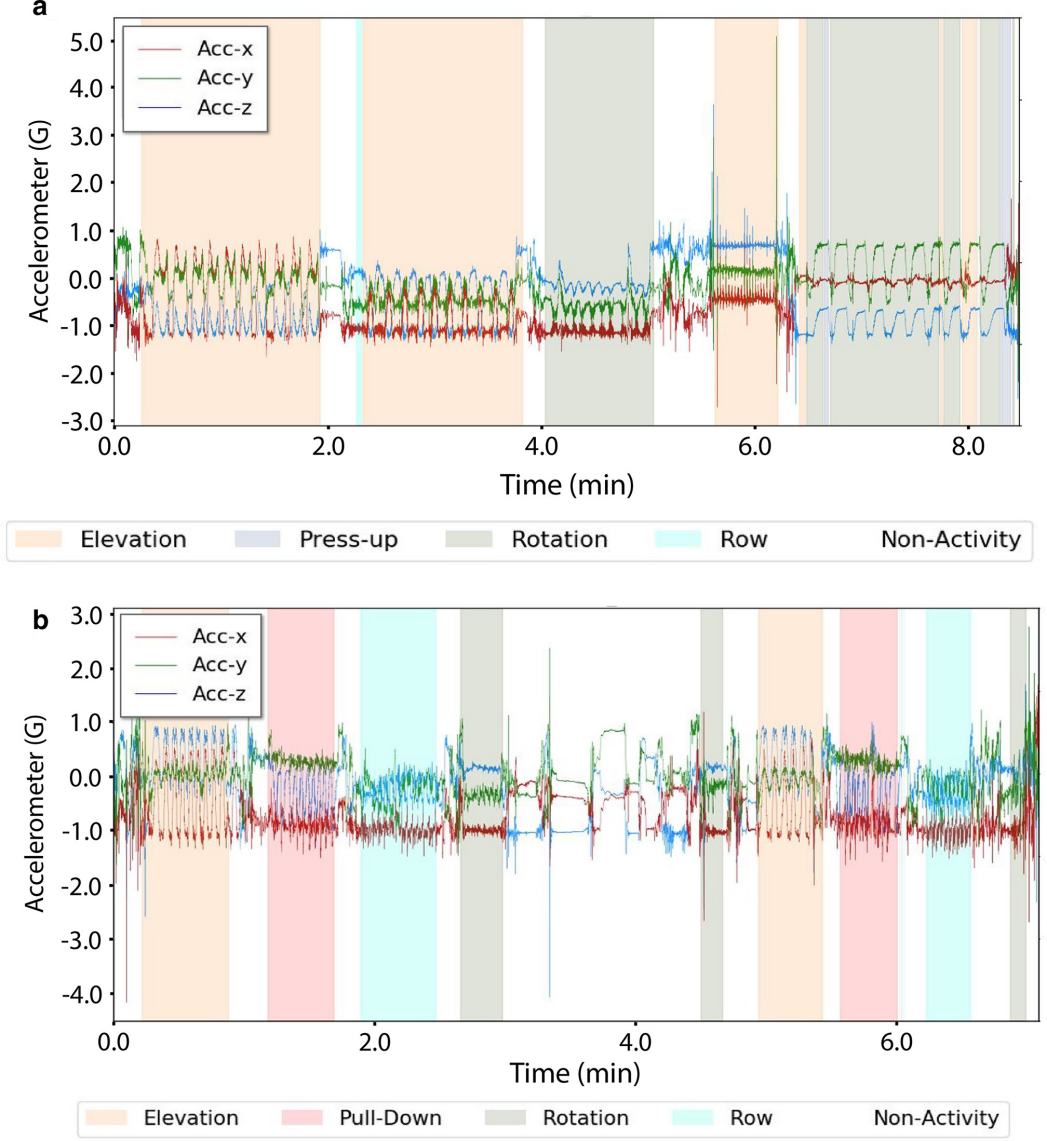
Examples of out-of-distribution detection and in-distribution classification on record from patients a) P1 and b) P4 with fully convolutional network supervised with proxy in training, grouped by simple motion category.

Figures 6a and 6b illustrate examples of ML pipeline output following OOD detection and in-distribution classification.

## Discussion

Patient-specific participation measures generated by our system have been shown to be correlated to improvements in patient outcomes (pain and disability).^24^ However, patients often deviate in unexpected ways from their prescribed physiotherapy when at home, potentially impacting the success of their treatment programme.^11,33-35^ Most IMU-based rehabilitation monitoring solutions validate algorithm performance solely on labelled data collected in a supervised clinic or laboratory setting.^21,28,36^ Confirming algorithm performance outside laboratory/clinical settings increases confidence for use in measuring at-home participation.

There is often greater variability in exercise technique among a patient population compared to healthy subjects. Variance in pain and restricted movement manifest in a wide range of motion, speed, smoothness, motor control, and intensity of exercise, which elevates the challenge of accurate ML exercise classification. Patients may also perform exercises incorrectly at home, yielding substantially different sensor recordings versus in-clinic supervised data or expected norms, leading to a higher likelihood of ML misclassification.

Accurate monitoring of physiotherapy performance is essential, as this information may ultimately be incorporated into the patient medical record and influence care decisions. Patients may be demotivated by system or algorithm issues, resulting in reduced participation scores.^24^ While small differences in AUROC may have a negligible impact on system performance for inaccurate algorithms, the impact on error rate becomes more pronounced as algorithm accuracy is improved (e.g. improving accuracy from 98% to 99% halves error rate). Furthermore, small differences in overall algorithm performance may reflect large differences in accuracy achieved for individual patients.

Our investigation indicates that exercise performance may deviate substantially comparing in-clinic and at-home settings for many patients. While patient P0 performed exercises very similarly in both settings, data collected in clinic and at home differed for P2 and P3. We also observed records with hours of continuous data collection with periods of very little movement (Figure 5), highlighting the importance of robust OOD detection.

First-pass OOD prediction on the at-home dataset yielded numerous Type I and Type II errors. Including a proxy OOD class in training eliminated many of these errors. At-home results were similar to the labelled in-clinic analysis, indicating that Spars9x-OOD adequately simulates at-home OOD data for the investigated records.

Improvements in OOD prediction in the patient-specific approach when proxy data are not included in training demonstrate the value of leveraging sessional data. Narrowing training data to the subset of exercises performed by a patient in their most recent supervised session avoids the influence of large variances in exercise technique among all subjects. This approach also adapts to changes in exercise technique over the course of recovery. Furthermore, because exercises are assigned by physiotherapists based on specific patient needs, some exercises are assigned more often, leading to balance issues. This a major advantage of the patient-specific method, where the KNN is trained on a smaller subset of exercises. However, since the patient-specific method is dependent on a small support set, it is particularly susceptible to labelling errors. In Supplementary Figure a, inertial data patterns for resisted shoulder internal rotation do not resemble other examples of this exercise in the dataset, indicating a likely labelling or collection error. Other records exhibited rest periods between exercise repetitions and/or non-exercise periods at the beginning/end of an exercise set, which are labelled as exercise and segmented. Ideally, such non-exercise periods should be filtered out. While the patient-specific approach implicitly adapts to changes in distributions over subsequent sessions through retraining of the KNN algorithm, a comparison with domain adaptation techniques would be of interest for future work as these may be less susceptible to the data issues mentioned.

Recently, we evaluated OOD detection methodology common in the image domain on human activity recognition (HAR) datasets.^26^ A key finding was that engineered statistical features appear superior for OOD detection in some HAR datasets versus DL feature extraction, whereas for in-distribution classification DL features these usually performed better. These findings are generally supported by this in-clinic OOD and in-distribution analysis.

Grouping classes improves classification accuracy because exercises with similar motions produce similar data patterns. Mean in-distribution accuracy grouped by exercise classes was 0.701. Reducing class number from 18 to 6 yields an expected accuracy of 0.751 based on random chance. Grouping by simple motion achieves a mean accuracy of 0.874, indicating accuracy gain due to exercise similarity. In contrast, grouping did not consistently improve OOD prediction. Although not of benefit in this study, grouping similar classes will result in differing DL features and feature extractors that could yield non-intuitive advantages in OOD prediction. Thus, choice of exercise grouping for OOD detection may depend on the investigated dataset and algorithms.

Results of some engineered feature algorithms in Table II and Table III are identical regardless of grouping. This result is specific to OOD prediction using classical models and engineered features (e.g. KNN in patient-specific) where grouping is washed out when converted to OOD, as this is ultimately a binary prediction problem. One exception is KMeans, where redefining classes will change cluster centre locations, so samples may be assigned different labels.

Time-consuming manual segmentation was conducted on at-home data by only a single rater and was limited to 25 records. Ground truth labels were assigned to the unlabelled dataset after unsupervised data collection, resulting in uncertainty. For example, in Supplementary Figure b, visual recognition of exercise was challenging and yielded ML predicted labels opposite manual segmentation labels. However, the algorithm is clearly incorrect in other records from this patient, where no confusion exists regarding the validity of the manual segmentation. In the future, an interactive app could be used by patients in combination with the smartwatch to indicate when they are performing their at-home exercises, yielding a labelled dataset. The implication of the low sample size and manual labelling of at-home inertial data is a greater uncertainty in the results of this portion of the study.

Missing data occurred when user or technical error resulted in unrecorded physiotherapy data or a poor-quality recording. These errors occurred in approximately 4% of data collections. Since reported algorithm accuracy neglects unrecorded therapy sessions, the overall system accuracy is overestimated.

A sensor re-sampling rate of 50 Hz was sufficient to accurately classify exercises performed by the rotator cuff patient population in this study, which performed repetitions relatively slowly (mean 3.8 seconds (standard deviation 2.3), right-skewed). Classifier performance could be degraded for other populations performing exercises more rapidly. To date, we have not specifically investigated the effect of re-sampling rate on classifier performance or its relationship to rate of exercise repetition.

Our system collects inertial data from a single IMU in a commercial smartwatch worn during shoulder physiotherapy exercise performance. This facilitated at-home use of the system, and qualitative data suggested a high degree of compliance in wearing the watch when performing exercises.^24^ However, algorithm performance is dependent on sufficient sensor motion, and press-up exercises exhibit lower magnitude motion at this site. After excluding press-ups from support, the patient-specific approach more accurately removed non-exercise periods. However, sensitivity decreased, likely due to difficulty identifying press-up exercises. While multiple sensors or differing sensor placement could ameliorate these issues, the ease of use and acceptability of the smartwatch motivate optimization of the current system workflow and analysis of this dataset.

Future research could combine supervised training using a proxy OOD dataset with other OOD detection techniques to potentially obtain an even more robust OOD identifier. Additionally, analysis on a larger patient dataset (collection currently ongoing) may yield a greater distinction between methods.

In summary, this study presented a ML pipeline for detecting and classifying in-clinic and at-home shoulder physiotherapy exercises, highlighting the use of a proxy dataset for accurate OOD detection. Leveraging patient-specific data improves performance but is impacted by data quality. ML algorithm selection is key to an objective IMU-based system for tracking patient at-home adherence. Deploying physiotherapy adherence monitoring systems requires ML algorithms to be validated in their ultimate use environment.
